# Fluorescence-Based Strategies to Investigate the Structure and Dynamics of Aptamer-Ligand Complexes

**DOI:** 10.3389/fchem.2016.00033

**Published:** 2016-08-03

**Authors:** Cibran Perez-Gonzalez, Daniel A. Lafontaine, J. Carlos Penedo

**Affiliations:** ^1^Laboratory for Biophysics and Biomolecular Dynamics, SUPA School of Physics and Astronomy, University of St. AndrewsSt Andrews, UK; ^2^RNA Group, Department of Biology, Faculty of Science, Université de SherbrookeSherbrooke, QC, Canada; ^3^Laboratory for Biophysics and Biomolecular Dynamics, Biomedical Sciences Research Complex, School of Biology, University of St. AndrewsSt. Andrews, UK

**Keywords:** fluorescence, 2-aminopurine, forster resonance energy transfer (FRET), single-molecule microscopy, aptamer dynamics

## Abstract

In addition to the helical nature of double-stranded DNA and RNA, single-stranded oligonucleotides can arrange themselves into tridimensional structures containing loops, bulges, internal hairpins and many other motifs. This ability has been used for more than two decades to generate oligonucleotide sequences, so-called aptamers, that can recognize certain metabolites with high affinity and specificity. More recently, this library of artificially-generated nucleic acid aptamers has been expanded by the discovery that naturally occurring RNA sequences control bacterial gene expression in response to cellular concentration of a given metabolite. The application of fluorescence methods has been pivotal to characterize in detail the structure and dynamics of these aptamer-ligand complexes in solution. This is mostly due to the intrinsic high sensitivity of fluorescence methods and also to significant improvements in solid-phase synthesis, post-synthetic labeling strategies and optical instrumentation that took place during the last decade. In this work, we provide an overview of the most widely employed fluorescence methods to investigate aptamer structure and function by describing the use of aptamers labeled with a single dye in fluorescence quenching and anisotropy assays. The use of 2-aminopurine as a fluorescent analog of adenine to monitor local changes in structure and fluorescence resonance energy transfer (FRET) to follow long-range conformational changes is also covered in detail. The last part of the review is dedicated to the application of fluorescence techniques based on single-molecule microscopy, a technique that has revolutionized our understanding of nucleic acid structure and dynamics. We finally describe the advantages of monitoring ligand-binding and conformational changes, one molecule at a time, to decipher the complexity of regulatory aptamers and summarize the emerging folding and ligand-binding models arising from the application of these single-molecule FRET microscopy techniques.

## Introduction

The ability of nucleic acid sequences to perform intermolecular interactions allows these molecules to adopt a range of tertiary structures enabling a variety of functions to be achieved (Saenger, 1984; Vieregg, 2010). Thus, in addition to the well-known activity of nucleic acids as storage and transporters of genetic information, more challenging protein-like functions have been unveiled in the last decades. For instance, the discovery that RNA can catalyze chemical reactions (Strobel and Cochrane, 2007) and regulate gene expression (Blouin et al., 2009a; Morris and Mattick, 2014; Watcher, 2014) has contribute to place “functional nucleic acids” not only as a structures to understand but also as molecules to exploit for biological and analytical applications (Li and Lu, 2009; Liu et al., 2009; Mulhbacher et al., 2010b; Radom et al., 2013). In particular, nucleic acid aptamers selected *in vitro* by systematic evolution of ligands by exponential enrichment (SELEX) to specifically recognize certain metabolites have revolutionized the field of molecular recognition (Stoltenburg et al., 2007; Mayer, 2009; Darmostuk et al., 2015). Although, nucleic acids only use four nucleotides compared to the 20 amino acids used by proteins, they recognize their cognate ligands with high affinity and selectivity mostly by altering the tertiary structure. Interestingly, counterparts of these *in vitro* selected RNA aptamers, so called riboswitches, were only discovered more than a decade later. Such RNA switches exhibit an extensive presence in nature as part of gene regulatory systems (Nahvi et al., 2002; Sudarsan et al., 2003; Winkler et al., 2003).

Since these early reports on naturally-occurring RNA aptamer sequences, a continuously growing number of natural aptamers have been discovered recognizing a variety of small metabolites (Henkin, 2008; Serganov and Nudler, 2013) including purine nucleotides and their derivatives (Batey et al., 2004; Mandal and Breaker, 2004), amino-acids such as lysine (Sudarsan et al., 2003), and glycine (Mandal et al., 2004), phosphorylated sugars such as D-glucosamine-6-phosphate (Winkler et al., 2004), protein cofactors including cobalamin (Nahvi et al., 2002), flavin mononucleotide (FMN) (Winkler et al., 2002a,b), thiamine-pyrophosphate (TPP) (Mironov et al., 2002), S-adenosyl-methionine (SAM) (Epshtein et al., 2003; Winkler et al., 2003) and some inorganic ligands including Mg^2+^ (Cromie et al., 2006; Dann et al., 2007; Ramesh and Winkler, 2010) and fluoride ions (Baker et al., 2012). Riboswitches are commonly found in the 5′-untranslated region of the messenger RNA and a typical riboswitch consists of two parts: the aptamer domain that recognize the ligand and the expression platform that interfaces with the regulating machinery and signals the ligand binding event (Blouin et al., 2009a; Garst et al., 2011; Serganov and Nudler, 2013). The riboswitch regulatory process is modulated by the presence of two mutually exclusive structures that differentially control genetic expression. In the last few years, synthetic riboswitches have been engineered by merging RNA-based sensing domains with regulatory domains and we refer to several excellent reviews for more detailed information (Groher and Suess, 2014). Also, because riboswitches are widespread in bacterial organisms but only one, the TPP riboswitch, has been found in plants or higher organisms, they have been proposed as attractive anti-microbial targets (Blount and Breaker, 2006; Mulhbacher et al., 2010a). For instance, it has been shown using a mouse model that a pyrimidine compound (PC1) that targets a guanine-binding riboswitch exhibits bactericidal activity and reduces *Staphylococcus aureus* infection in mammary glands (Mulhbacher et al., 2010a). Very recently, a synthetic mimic of FMN, ribocil, that efficiently inhibits FMN-mediated gene expression and inhibits bacterial growth has been reported (Howe et al., 2015). Notably, although ribocil is structurally different from the natural ligand, it shares enough contacts to the FMN binding site to exhibit high affinity and efficiently compete with the natural ligand for binding to the RNA aptamer.

### The importance of metal ions in aptamer function

The formation of a ligand-binding competent 3D structure depends on a number of variables including the sequence, the length of the nucleic acid and the environmental conditions (Patel et al., 1997; Sosnick and Pan, 2003; Draper, 2008; Leipply et al., 2009). Here, the presence of monovalent (Na^+^, K^+^) and divalent metal ions, specially Mg^2+^ ions, that act by screening the negative charge of the nucleic acid chain is known to play a critical role in the formation of the ligand-binding pocket and the stability of the aptamer-ligand complex (Draper, 2004; Woodson, 2005). In the presence of the targeted metabolite and in the appropriate environmental conditions (pH, ionic strength), the aptamer undergoes conformational changes driven by intermolecular interactions such as hydrogen bonding, base stacking and electrostatic interactions (Hermann and Patel, 2000; Schroeder et al., 2004). In addition to the screening of the negatively charged nucleic acid chain by so-called diffusive ions, it is not uncommon to find in the X-ray structures of aptamer-ligand complexes specifically positioned divalent metal ions (i.e., Mg^2+^) acting as bridges between nucleotides and forming critical bonds with the ligand (Sosnick and Pan, 2003). A remarkable example of Mg^2+^ ions actively participating in the formation of the ligand-binding pocket is exemplified by the naturally-occurring fluoride RNA aptamer that targets fluoride ion with a K_D_ of ~60 μM and discriminates against other halogen ions (Baker et al., 2012). The crystal structure of the fluoride aptamer from *Thermotoga petrophila* provides an atomic-level example of how nature has developed strategies so that a negatively charged nucleic acid polymer can efficiently recognize an anionic ligand (Ren et al., 2012). The structure revealed a fluoride ion encapsulated within the RNA core and anchored in place by an inner shell of three Mg^2+^ions octahedrally coordinated to water molecules and an outer shell of phosphates (Ren et al., 2012).

### Structural diversity and modularity in nucleic acid aptamers

Typically, structural motifs found in nucleic acid aptamers include stem, loops, bulges, k-turn structures, hairpins, and pseudoknots, as well as combinations of them organized in a modular manner. Perhaps, one of the most striking differences between natural and SELEX-generated RNA aptamers is that naturally-occurring aptamers exhibit more complex architectures than the single-aptamer/single-ligand configuration characteristic of *in vitro* selected molecules. For instance, the tetrahydrofolate-sensing (THF) aptamer has been shown by X-ray crystallography to accommodate two THF ligands within the aptamer structure and strong cooperative binding was demonstrated at physiological concentrations of Mg^2+^ ions (Trausch et al., 2011).

Another atypical organization of natural aptamers is exemplified by the sensing domain of the glycine-responsive riboswitch which is composed of two adjacent aptamers joined by a short linker and followed by a single expression platform (Mandal et al., 2004; Huang et al., 2010). The possibility that both aptamers were acting cooperatively to recognize both glycine ligands was confirmed by Hill coefficients of ~1.6 and 1.4 for *Vibrio cholerae* and *Bacillus subtilis gsvT* riboswitches, respectively (Mandal et al., 2004; Huang et al., 2010; Butler et al., 2011). It was proposed that such cooperative interaction would allow bacteria to have a more pronounced response to variations in glycine concentration, and as a result, rapidly activate or repress the genes encoding for the glycine cleavage system. However, two recent studies have identified an extended 5′ end that base pairs with the linker region and forms a stable k-turn motif (Kladwang et al., 2012; Sherman et al., 2012). This additional structural element, truncated in initial studies of the minimal riboswitch, has been shown to facilitate inter-aptamer interactions and increase binding affinity. Importantly, several studies have demonstrated that aptamer constructs containing this k-turn motif displayed no cooperativity (Baird and Ferré-D'Amaré, 2013; Esquiaqui et al., 2014), thus raising the question of what are the advantages of a tandem architecture that has preserved this configuration during evolution instead of being replaced by a single aptamer. Recent studies suggested that a dimeric structure is required for ligand binding to occur. While the formation of inter-aptamer contacts is thought to be favored by the presence of a highly stabilized P1 stem on aptamer-1, the dimerization and ligand binding ensures that the P1 stem of the adjacent aptamer becomes stabilized and acts as the switch for gene regulation (Baird and Ferré-D'Amaré, 2013).

### Improving SELEX-Generated structures using composite aptamers

The tandem organization and cooperative features described for the minimal glycine riboswitch aptamer have also been explored to develop artificial sequences where aptamers structures are organized in clusters to improve binding affinity (Hasegawa et al., 2016). One of the first examples of such composite architectures was demonstrated by connecting a 15-mer thrombin aptamer and a 29-mer thrombin aptamer (Hasegawa et al., 2008). The joined structure exhibited a sub-nanomolar binding with a 10-fold increase with respect to the single aptamer. It was subsequently shown that the 15-mer aptamer within the bivalent construct exhibited a 50-fold decreased off rate compared to that of the monovalent aptamer due to cooperative binding even though the on rate was similar (Kim et al., 2008). An important consideration in the design of these composite aptamers is the nature and flexibility of the linker region joining the aptamers (Bujotzek et al., 2011). For instance, Tian et al. have shown that when both, 15-mer and 29-mer, thrombin aptamers are joined by a flexible polyethylene glycol (PEG) linker, the affinity of this construct was almost two orders of magnitude higher than those of the individual aptamers (Tian and Heyduk, 2009). Importantly, the intrinsic conformational freedom of the linker adds a degree of uncertainty that requires experimental adjustment of the linker length to optimize the position of the aptamers in close proximity to their targeted sites (Hasegawa et al., 2008).

In addition to flexible linkers, double-stranded DNA and double-stranded RNA have also been used as rigid linkers that allow to modulate not only the distance between aptamers but also their relative orientation. In this context, a bivalent aptamer for heat shock factor 1 (HSF1) protein carrying a 12-bp dsRNA was shown to exhibit a 24-higher affinity and the incorporation of a 3-mer ssRNA sequence within the dsRNA region increased a further 5-fold the binding affinity (Zhao et al., 2013). This suggests that aptamer clusters using combinations of dsRNA and ssRNA regions in the linker sequence may offer opportunities to simultaneously optimize aptamer distance and orientation with respect to their target sites. In recent years, the aptamer-design approach has been complemented by a selection-based approach that includes the oligonucleotide linker. Using this evolution strategy bivalent aptamers consisting of two thrombin aptamers including a 35-mer randomized linker sequence, where most of the bases formed double helices joined by a short ssDNA region, displayed a 200-fold higher affinity than the 15-mer aptamer (Ahmad et al., 2012). The application of these selection methods is rapidly emerging as a useful approach to isolate composite aptamers with activity against a single target (Shi et al., 2007; Nonaka et al., 2010) or against distinct protein epitopes (Cho et al., 2015).

## Biophysical methods to investigate aptamer-ligand complexes

Quantifying key properties of aptamer-ligand interactions such as affinity, binding kinetics and specificity is critical to determine their technological relevance. However, it is interesting to note that despite the large number of aptamers reported to date, only a small subset of them has been characterized in detail. A number of biophysical techniques have been applied to determine binding kinetics and affinity values including stopped-flow fluorescence spectroscopy (Wickiser et al., 2005; Lang et al., 2007), equilibrium filtration (Huizenga and Szostak, 1995), gel-shift assays (Smith et al., 2009) and label-free methods such as isothermal titration calorimetry (ITC) (Lin et al., 2008; Sokoloski et al., 2012), kinetic ITC (Burnouf et al., 2012), surface-plasmon resonance (SPR) (Polonschii et al., 2010) and microfluidic SELEX (Tan et al., 2016). Selecting one of these methods depends on the specific aptamer under study and the required sensitivity as recently reviewed by DeRosa and coworkers (McKeague et al., 2015). Moreover, the specific nature of the aptamer-ligand interaction may also impose restrictions in the type of bioanalytical method that can be employed. For example, SPR has been used to characterize protein-aptamer interactions but their wide application to small molecule-aptamer interactions remained limited until recent years (Chang et al., 2014).

To have a sense of the broad range of binding affinities and kinetic rates that aptamer-ligand interactions can exhibit, it is worth to provide some benchmark limiting values that have been reported in the literature. Artificial aptamer sequences with affinities in the low picomolar range have been reported that bind, for example, to platelet derived growth factor B (PDGF-BB) (~28 pM) (Ahmad et al., 2011) and the human immunodeficiency virus (HIV) reverse transcriptase (~4 pM) (Ferreira-Bravo et al., 2015). Composite aptamers generated using an array-based approach with activity against VEGF-165 and very high binding affinities (~97 pM) have also been reported (Cho et al., 2015). Most natural aptamers display relatively moderate affinities compared to artificial aptamers, but very tight binding has also been observed for the c-di-GMP class I aptamer that, in fact, exhibits the tightest reported K_D_ for an RNA-small molecule interaction (~10 pM) and the TPP-sensing *Thi1* riboswitch aptamer (~200 pM) (Welz and Breaker, 2007). At the other end of the affinity bracket we have many examples of artificial and natural aptamers such as the arginine and citrulline aptamers with K_D_ values in the range 50–70 μM (Chang et al., 2014) and the glycine riboswitch aptamer (~20–80 μM) (Mandal et al., 2004). Usually, the association rates are slower than diffusion limited and values between 10^4^–10^5^ M^−1^ s^−1^ have been obtained for most aptamers. Dissociation rates found for most complexes between small molecules and aptamers were in the range between 10^−2^ and 10^−5^ s^−1^ (Chang et al., 2014) and in general, natural aptamers normally have slower dissociation rates (~10^−3^ s^−1^) than artificial aptamers (~10^−2^–10^−3^ s^−1^). In the context of natural aptamers, it is interesting to note that even closely related sequences such as the class I and class II c-di-GMP aptamers, can exhibit very different off rates. Comparing both c-di-GMP classes, the class II aptamer, with a 60-fold higher affinity than the class I aptamer, has a 7-fold higher association rate and >400-fold higher dissociation rate. This was interpreted as evidence of how regulatory aptamers may be fine-tuned to induce a different biological response to the same small-molecule input (Smith et al., 2011).

In addition to the thermodynamic and kinetic characterization of the aptamer-ligand interaction, the specificity of SELEX-engineered and natural aptamers relies on achieving a three-dimensional structure capable of accommodating the ligand whilst allowing efficient discrimination against structurally close analogs. A range of biophysical and biochemical methods have been employed to investigate how the interplay between RNA folding and ligand recognition mechanisms enable the formation of these highly specific contacts. For instance, a number of chemical and enzymatic methods have been developed to probe the conformation of the RNA sequence including in-line probing, hydroxyl radical probing, nucleotide interference mapping (NAIM) and selective 2′-hydroxyl acylation analyzed by primer extension (SHAPE) to mention some of them. These methods can provide information about local variations of the secondary structure taking place during folding or ligand binding (Merino et al., 2005; Peng et al., 2012). A detailed analysis of the application of these methods is beyond our scope and we refer the reader to excellent reports recently published for chemical probing in general (Regulski and Breaker, 2008; Weeks, 2010) and those describing more specific method such as SHAPE (Low and Weeks, 2010).

Biophysical techniques that can extract information about the global architecture of the RNA such as nuclear magnetic resonance (NMR) and X-ray crystallography have been pivotal to understand the function of nucleic aptamers (Felden, 2007). Traditionally, X-ray crystallography of RNA has been considered to be more challenging than for proteins, mostly because of the difficulties in rapidly purifying large amounts of RNA and the lack of methods to solve the “phasing problem” once diffracting crystals are obtained (Reyes et al., 2009). While protein crystallography was transformed by the development of affinity purification techniques and seleno-methionine labeling, similar general strategies for RNA still lack the general applicability of their protein counterparts. Nevertheless, several recent advances in “directed soaking” and specific positioning of heavy atoms using cobalt (III) hexammine, iridium (III) hexamine or Os (III) hexamine ions have resulted in a significant increase, during the last decade, in the number and, more importantly, the complexity of solved RNA structures (Felden, 2007; Keel et al., 2007). Whilst at the beginning of the last decade only a handful of RNA structures were available including tRNA, the hammerhead ribozyme and fragments of the group I ribozyme, nowadays the X-ray structure of almost all known natural aptamers has been solved (reviewed in Serganov, 2010; Serganov and Patel, 2012a,b). Interestingly, the majority of these high-resolution X-ray structures correspond to the ligand-bound state and X-ray crystal structures for the ligand-free state have only been solved for representatives of the preQ1 (Jenkins et al., 2011), S-adenosylmethionine (Stoddard et al., 2010), and lysine (Garst et al., 2008; Serganov et al., 2008) aptamer classes. These ligand-free crystal structures suggest that, even in the absence of ligand, the aptamer can adopt a conformation very similar to that of the ligand-bound state and raises the question of how the ligand can access its binding pocket. However, it has been suggested that, in most cases, the ligand-free X-ray structure may represent only a minor population of the ensemble present in solution (Liberman and Wedekind, 2012).

Due to the limitations of X-ray crystallographic methods to provide a comparative picture of aptamer structures with and without ligand, considerable efforts have been put in using NMR as a technique to extract structural information in solution from both states (Latham et al., 2005; Bothe et al., 2011; Dominguez et al., 2011). For instance, improvements in strategies for isotopic labeling that allow the production of ^13^C and ^15^N-labeled RNAs (Lu et al., 2010; Duss et al., 2012) and advances in the acquisition and analysis of residual dipolar couplings (MacDonald and Lu, 2002; Lipsitz and Tjandra, 2004) have allowed to obtain high-resolution solution structures for a variety of synthetic (Feigon et al., 1996; Puglisi and Williamson, 1999) and natural aptamers (Noeske et al., 2005; Buck et al., 2009). Recent advances in real-time NMR data collection have enabled, for example, to monitor the conformational changes following the transition between the ligand-free and the ligand-bound forms of the adenine riboswitch (Lee et al., 2010).

In addition to X-ray crystallography and NMR techniques, improvements in solid-phase synthesis of modified oligonucleotides (Verma and Eckstein, 1998; Höbartner and Wachowius, 2010), together with the use of fluorescent nucleotide analogs (Jameson and Eccleston, 1997; Cremo, 2003) and more efficient post-synthetic labeling strategies (Rublack et al., 2011), has allowed a remarkable increase in the application of fluorescence-based methods to characterize aptamer-ligand interactions. In particular, the emergence of fluorescence microscopy techniques that allow monitoring the dynamics of individual structures has revolutionized our understanding of nucleic acid function and structure (McCluskey et al., 2014; Boudreault et al., 2015; Perez-Gonzalez and Penedo, 2015). In this review we will focus on those techniques that rely on the labeling of the aptamer sequence with one or two fluorophores, or a fluorophore and a quencher to characterize aptamer function at ensemble level. In the last part of the review, we will describe the use of single-molecule microscopy techniques, mostly in FRET format, to monitor the conformational dynamics of single aptamers.

### Fluorescence strategies to monitor aptamer function using singly-labeled nucleic acid sequences

The rationale behind the use of aptamers labeled with a single fluorophore is based on the fact that the photophysical properties of fluorescent molecules (i.e., emission intensity, absorption and/or emission wavelength, fluorescence lifetime, anisotropy) are known to be very sensitive to the local environment surrounding the fluorescent probe (Cho et al., 2005; Klymchenko and Mely, 2013; Siraj et al., 2016). Thus, covalent attachment of a single dye to an aptamer sequence at a position that can experience substantial structural changes during ligand binding should alter the electronic environment of the probe and result in a measurable variation in one, or more, of the emission properties of the attached fluorophore. In the last two decades, many strategies have been developed to exploit this effect and signaling aptamers carrying one or two fluorophores have been constructed to signal ligand binding (Nutiu and Li, 2005; Wang et al., 2011; Arora et al., 2015; Li et al., 2015) (Table 1). Obviously, labeling aptamers with a fluorophore may influence the nucleic acid conformation or alter the binding affinity and the appropriate controls should be carried out to rule out this possibility using for instance, circular dichroism, or chemical probing techniques (Blouin et al., 2009a; Juskowiak, 2011).

**Table 1:** **Summary of the most relevant dyes and fluorescence strategies referred in the manuscript**.

	**Fluorescence Technique**	**Specific dye**	**Representative Example**	**References**
**Single dye**	Intensity-based	Acridine	Adenosine aptamer	Jhaveri et al., 2000
		CdSe/ZnS Quantum dots	*E. coli* O111:B4 bacteria	Dwarakanath et al., 2004
		Ethidium bromide	Thrombin aptamer	Li B. et al., 2007
		Fluorescein	Adenosine aptamer	Jhaveri et al., 2000
		OliGreen	ATP aptamer	Huang and Chang, 2008
		Pbs Quantum dots	Thrombin aptamer	Choi et al., 2006
		Pyrene derivative	ATP aptamer	Yamana et al., 2003
		Ru[(phen)2(dppz)]2+	IgE, thrombin and ATP aptamers	Jiang et al., 2004; Wang et al., 2005
		TOTO	PDGF-BB aptamer	Zhou et al., 2006
		YOYO	Thrombin aptamer	Joseph et al., 1996
	Anisotropy-based	5-carboxytetramethylrhodamine	Tobramycin RNA aptamer	Wang et al., 1996
	Nucleotide analogs	2-Aminopurine	Adenine, SAM-I/SAM-II and preQ1-II aptamers	Lemay et al., 2006; Heppell et al., 2011; Soulière et al., 2011
		4-amino-6-methylpteridone	Thrombin aptamer	Katilius et al., 2006
		Pyrrolo-dC	HIV-1 polypurine extract	Dash et al., 2004
**Doubly-labeled**	Molecular beacons	FAM-Dabcyl	Thrombin aptamer	Li et al., 2002
		Pyrrolo-dC-7AAMD	Various DNA hairpins	Zhang and Wadkins, 2009
		Sulforhodamine-dinitroaniline	*E.coli* SBR-2 RNA aptamer	Sunbul and Jäschke, 2013; Arora et al., 2015
	FRET-based beacons	Coumarin-FAM	Thrombin aptamer	Li et al., 2002
		Cy3-Cy5	Polymerase II transcriptional activity	Shin et al., 2014
	FAM-TMR	Angiogenin	Li et al., 2008
	Dimers	Pyrene excimer	PDGF, thrombin and ATP aptamers	Yang et al., 2005; Zheng et al., 2015

*Representative examples using each dye and fluorescence technique together with the appropriate reference are also given*.

#### Intensity-based signaling aptamers

**Figure 1:**
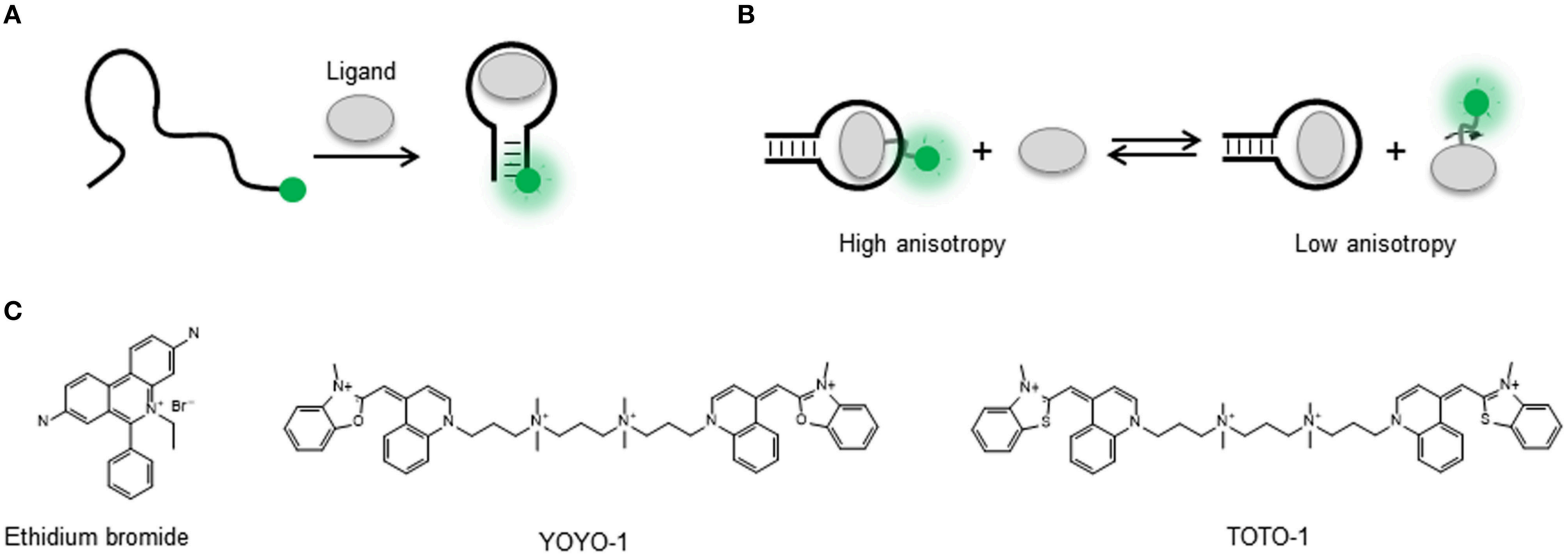
**(A)** Intensity-based signaling strategy for the detection of conformational changes in a singly-labeled aptamer upon ligand binding. A single dye can probe local changes in the nucleic acid structure induced by target binding and as result experience an increase or decrease in the fluorescence emission. **(B)** Fluorescence anisotropy-based signaling aptamer strategy in a ligand competition assay. A fluorescently labeled ligand bound to its target sequence undergoes a slower tumbling motion (high anisotropy) than in the unbound state (low anisotropy). In the presence of non-labeled ligand, displacement of the fluorescent ligand results in a transition from high to low anisotropy. **(C)** Molecular structure of the nucleic acid intercalators ethidium bromide, YOYO-1, and TOTO-1.

The use of covalently attached fluorophores that signal ligand binding through an increase in emission intensity (Figure 1A) is exemplified by two anti-adenosine (ATP) aptamers carrying either an internal acridine or a fluorescein molecule at the 5′ end (Huizenga and Szostak, 1995; Jhaveri et al., 2000). For the acridine-labeled aptamer, an increase in fluorescence emission was observed in the presence of ATP but not GTP and two close dissociation constants (K_D1_~ 30 μM and K_D2_ ~ 50 μM), confirming that the labeled construct is able to discriminate between nucleotides and bind two ATP molecules as observed for the unlabeled aptamer. In another study, Yamana et al. designed a pyrene derivative and attached it to various positions in an ATP-responsive DNA aptamer (Yamana et al., 2003). They obtained an emission increase for only one of the positions tested as a function of ATP. When the pyrene derivative was incorporated at the 2′-ribose position, a fluorescence increase was also observed for some of the positions tested. Merino and Weeks explored further the sensitivity of the 2′-ribose position to changes in the surrounding electrostatic environment caused by variations in the flexibility of the nucleotide during ligand binding (Merino and Weeks, 2003). A single 2′amino cytosine incorporated at specific positions in the adenosine monophosphate (AMP), tyrosinamide and argininamide aptamers was used to fuse a BodipyFL fluorophore. All the fluorescently labeled aptamers showed good discrimination between the natural and non-cognate ligands such as cytosine monophosphate (CMP), alaninamide, and lysinamide. The fluorescence increase observed for those positions that were found to respond to ligand binding was between 2- and 4-fold. A similar approach, but replacing the organic fluorophore by a semiconductor nanocrystal (quantum dot or QD), was also developed to monitor the binding of a DNA aptamer conjugated with the QD to bacteria (Dwarakanath et al., 2004). The QD exhibited two fluorescent peaks in the unbound state at 600 and 400 nm, but upon binding to *E. coli* the peak at 600 nm disappeared and the 400 nm peak exhibited an intensity increase. The authors suggested that this variation in relative intensity could be due to changes in local environment such as pH or electric charge but this needs further investigation. In another example, a thrombin aptamer-functionalized PbS QD showed quenching of the QD emission in the presence of thrombin and a detection limit of 1 nM (Choi et al., 2006; Deng et al., 2014). Subsequent applications of the QD-aptamer concept for specific labeling of tumor cells and to detect pathogens have been recently reviewed (Chiu and Huang, 2009; Wang et al., 2011; Gedi and Kim, 2014).

Strategies that do not require covalent attachment but involve the use of dyes such as ethidium bromide, YOYO, and TOTO, that either intercalate between the base pairs or bind the grooves of the nucleic acid helix, have also been applied for aptamer signaling (Liu and Lu, 2005; Sarpong and Datta, 2012; Figure 1C). The basic principle is that in the unbound state these dyes exhibit negligible fluorescence emission but they undergo a very strong fluorescence enhancement when bound to the nucleic acid structure. Interestingly, most applications have used a decrease in the dye emission induced by variations in the amount of bound fluorophore to monitor aptamer structural rearrangements upon ligand binding. For example, a very early study used YOYO staining of a 15-mer thrombin DNA aptamer to detect thrombin binding (Joseph et al., 1996). In a more recent study, the TOTO dye was used with a DNA aptamer against growth factor B chain. In this case, a fluorescence decrease was observed in the presence of the target with a detection limit of 0.1 nM and high selectivity (Zhou et al., 2006). The formation of guanine quadruplexes (GQ in the presence of K^+^ ions, known to stabilize the quadruplex structure, was monitored using OliGreen, a fluorophore that binds to single-stranded DNA (Huang and Chang, 2008). In the presence of K^+^, the formation of the GC induced the release of OliGreen, resulting in a decrease in fluorescence intensity. Inorganic metal complexes carrying the dppz intercalator, such as a Ru[(phen)_2_(dppz)]^2+^, displayed weak luminescence in buffers and a significant increase upon binding to IgE, thrombin and ATP aptamers (Jiang et al., 2004; Wang et al., 2005). The detection limits observed were 0.1, 0.01, and 1 nM, respectively. These values are much lower than those obtained by other detection methods and it was suggested that the metal complex remained bound to the aptamer after ligand binding. A more general method to detect target binding was developed by Li et al. using an ethidium bromide stained cDNA hybridized to the thrombin aptamer (Li B. et al., 2007). Thrombin binding was detected by the release of the cDNA and the subsequent decrease in fluorescence due to the ethidium bromide becoming released to the solution. In general, the use of groove-binders and intercalator dyes in aptamer sensing offers the advantage of being a label-free approach, as no modification needs to be introduced in the oligonucleotide sequence. However, as broader application of these methods is compromised by the uncertainty on the exact positioning of the fluorophores on the aptamer sequence and by the fact that so far most of them have used quenching to report target detection. The former makes difficult to generate strategies for further optimization, and the latter makes these assays prone to false positives by quenchers present in the environment (Liu et al., 2009).

#### Anisotropy-based signaling aptamers

In addition to the number of photons emitted by a fluorophore under continuous illumination, the use of fluorescence species as biomolecular probes offers the advantage that additional observables such as the degree of polarization of the emitted light (anisotropy) and the fluorescence lifetime are also available from the same covalently attached dye. In contrast to fluorescence intensity that can be affected by environmental factors such as background fluorescence and bleaching, fluorescence anisotropy and fluorescence lifetime are almost independent of these interferences (Liu et al., 2009). Fluorescence anisotropy, in particular, has been used widely employed to study numerous biomolecular interactions because it does not require the expensive pulsed lasers and fast detectors needed to acquire fluorescence lifetimes and it can be recorded in a conventional steady-state fluorimeter additionally equipped with polarizers (Jameson and Croney, 2003; Yan and Marriot, 2003; Hall et al., 2016). Fluorescence anisotropy is a measure of the ability of a fluorophore to produce depolarized emission when excited with polarized light. Small organic fluorophores rotate fast in solution during their lifetime in the excited state, typically a few nanoseconds, and therefore they depolarize light very efficiently (Lakowicz, 1999; Siraj et al., 2016). The photons emitted from organic dyes in solution preserve very little of the polarization with the light they were excited and as a result they show a low anisotropy value. In contrast, when a fluorophore or a biomolecule associates to another biomolecule, the rotational motion of the complex is much slower and the fluorophore is not able to depolarize light so efficiently (Figure 1B). As a result, a high anisotropy will be observed. In order to have a large anisotropy change when the complex forms, the fluorophore is normally positioned in the partner with the lowest molecular weight (Liu et al., 2009; Gradinaru et al., 2010).

In the context of aptamer function, an increase in anisotropy has been employed to detect the binding of an aptamer to a dye-labeled aminoglycoside antibiotic, tobramycin (Wang et al., 1996; Llano-Sotelo and Chow, 1999). The same aptamer was also tested using a competitive anisotropy assay (Figure 1C) where the dye-labeled tobramycin-aptamer complex was mixed with unlabeled tobramycin. As a result of competitive binding and release of the labeled tobramycin, a decrease in anisotropy was observed. When the target is a protein instead of a small molecule, the fluorescence dye is usually placed in the aptamer to maximize the change in anisotropy (Liu et al., 2009). This method has been applied to study the thrombin (Potyrailo et al., 1998) and PDGF aptamers (Fang et al., 2001) and aptamers for amyloid-β peptides (Ylera et al., 2002; Takahashi et al., 2009), angiogenin (Li W. et al., 2007), and Hg^2+^ (Ye and Yin, 2008). The level of detail that can be obtained about the aptamer-protein interaction using fluorescence anisotropy is exemplified by the work from Zhang et al. (2011, 2012). Their work demonstrates that fluorescence anisotropy can be used to exquisitely map the interaction between the aptamer and the target protein at single nucleotide level (Zhang et al., 2011), which can be useful for the design of improved aptamers for analytical applications. Fluorescence anisotropy has been employed also to develop aptamer-based homogeneous assays (Sassolas et al., 2011) and for high-throughput screening (Weng et al., 2012). For instance, McCauley et al. were able to detect four different proteins using a chip-based array containing four aptamers (McCauley et al., 2003) and the Famulok group demonstrated the potential of aptamer-based anisotropy sensors for the detection of inhibitors for the cytohesin class of small guanosine exchange factors (Hafner et al., 2006, 2008).

#### 2-aminopurine and other fluorescent nucleotide analogs

As described in the previous sections, it is possible to design aptamer sensors based on changes in fluorescence intensity or anisotropy from a fluorophore strategically positioned within the aptamer sequence. An alternative approach that avoids the use of bulky fluorophores consists in the incorporation of fluorescent nucleotide analogs that retain the ability to form hydrogen bonds and preserve the self-recognition properties of natural nucleobases (Wilhelmsson, 2010). A demonstration of this concept was provided by Katilius and coworkers who replaced the thymine base at position 7 of the 15-mer thrombin aptamer with the fluorescent nucleobase 4-amino-6-methylpteridone (Katilius et al., 2006). In contrast to the 4-fold increase in fluorescence observed for the same aptamer-ligand complex when using a covalently attached bulky fluorophore, they observed a 30-fold fluorescence enhancement using the nucleotide analog and the construct retained the wild-type binding affinity (K_D_ ~ 12 nM).

**Figure 2:**
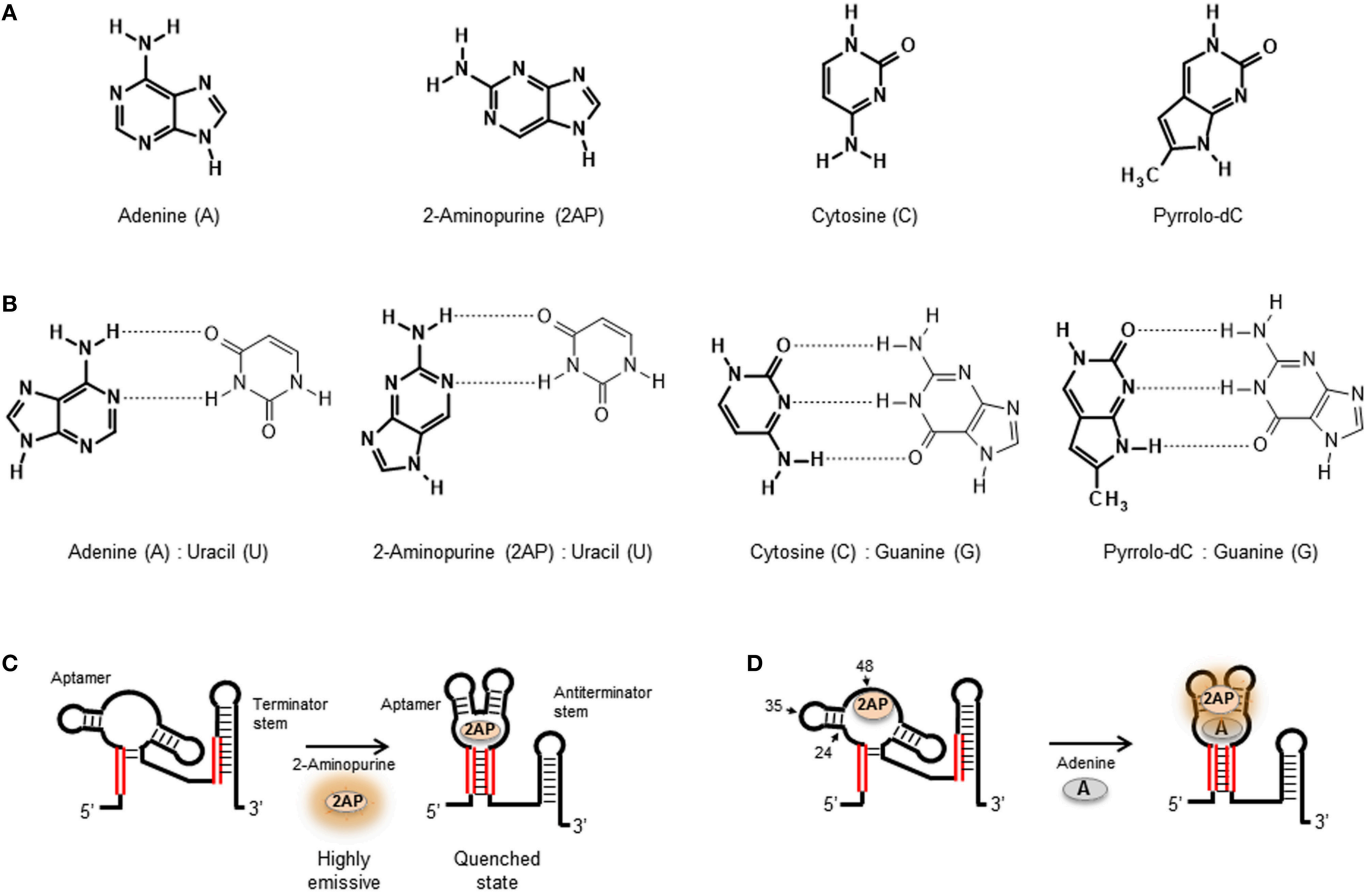
**(A)** Molecular structure of the natural nucleobases adenine and cytosine compared with their fluorescent analogs 2-aminopurine (2AP) and Pyrrolo-dC, respectively. **(B)** Base-pair interactions of adenine, 2AP, cytosine, and Pyrrolo-dC in RNA structures. **(C)** 2AP mimics the natural adenine ligand and binds the adenine riboswitch aptamer similar affinity. The high quantum yield 2AP free in solution is quenched when bound to the aptamer sequence. **(D)** Substitution of adenine by 2-aminopurine within the natural oligonucleotide sequence enables to probe local changes in aptamer structure induced by ligand binding. Representation of the U48AP construct of the adenine riboswitch and location of the A24AP and U35AP substitutions used in the work by Lemay et al. (2006) to monitor aptamer folding and ligand binding in the adenine riboswitch sensor domain. For the U48AP variant, the ligand binding induces a change of the aptamer structure into a conformation in which the 2AP is directed into the solvent, thus resulting in an increase in fluorescence emission.

Among the available fluorescent nucleotide analogs, 2-aminopurine (2AP) (Figure 2A) has been extensively used to investigate conformational changes in DNA and RNA and their interactions with proteins and large ribonucleopotein complexes (Rachofsky et al., 2001; Ilgu et al., 2013). 2-aminopurine is an analog of adenine that can form also stable base pairs with uracil (RNA) and thymine (DNA) (Figure 2B). The use of 2AP as a nucleic acid probe is very attractive because its photophysical properties, quantum yield, anisotropy, and fluorescence lifetime are extremely sensitive to the local conformation and the stacking geometry and nature of the flanking bases (Rachofsky et al., 2001). In general, the high quantum yield of 2AP in free solution (~0.68) is drastically quenched when 2AP is inserted in the nucleic acid sequence (Stivers, 1998). It has been shown that this quenching mechanism is mostly dominated by stacking interaction between 2AP and the adjacent bases with very little contribution from hydrogen bonding and base pairing (Hardman and Thompson, 2006). The sensitivity of 2AP to the stacking micro-environment has been used to monitor local re-arrangements in the nucleic acid structure such as base flipping and local melting process induced by nucleic acid folding (Rist and Marino, 2002), as in the case of natural aptamers (Ballin et al., 2007; St-Pierre et al., 2014), or induced by the action of nucleic acid processing enzymes (Hariharan and Reha-Krantz, 2005; Finger et al., 2013). In addition to 2AP, the non-natural nucleobase furano-dT has been used to generate *in-situ* a fluorescent derivative of cytosine, pyrrolocytosine (pyrrolo-dC) (Figures 2A,B), following ammonia treatment of the nucleotide sequence at the final stage of solid-phase synthesis (Berry et al., 2004). Pyrrolo-dC has been much less exploited than 2AP as a reporter of nucleic acid structure, and to date, only a handful of studies have been reported using pyrrolo-dC alone and in combination with 2AP in a molecular beacon format (Dash et al., 2004; Zhang and Wadkins, 2009).

With the exception of the adenine-responsive riboswitch aptamer for which it has been shown that the aptamer domain recognizes 2AP with similar affinity (K_D_ ~ 250 nM; Figure 2C) as the adenine ligand (Mandal and Breaker, 2004; Lemay et al., 2006; Lemay and Lafontaine, 2007), the majority of applications of 2AP have focused on monitoring changes in secondary and tertiary structure. However, in the absence of X-ray data, selecting appropriate positions within the nucleic acid structure to insert a 2AP substitution that can be sensitive to ligand binding or folding events can be a tedious and random task. Here, a strategy recently developed by Souliere et al. allows to identify positions to within the RNA sequence where the introduction of a 2AP substitution may act as a sensitive reporter of folding or ligand binding (Soulière et al., 2011; Soulière and Micura, 2014). The method uses SHAPE reactivity profiles to predict positions where the nucleotides are flexible because they are looped out or partially unstacked and differentiate them from those located in more constrained positions. SHAPE methods take advantage of the chemical reactivity between 2′ hydroxyl groups and electrophilic groups such as N-methylisatoic anhydride (NMIA) and benzoyl cyanide (BzCN) to form 2′-O-adducts (Wilkinson et al., 2006; Mortimer and Weeks, 2009). Using BzCN in three model riboswitch aptamers (adenine, SAM-II and PreQ1 class), a correlation was found between variations in SHAPE-reactivity profiles and 2AP fluorescence emission (Soulière et al., 2011).

2-aminopurine substitutions have been extensively used to investigate the folding and ligand-binding mechanisms of natural aptamers and this has been recently reviewed (St-Pierre et al., 2014). As an example, we will briefly discuss the use of 2AP to monitor local changes in nucleic acid structure in the context of purine-sensing aptamers (Figure 2C). Biochemical and structural data suggested that the *add* riboswitch aptamer from *Vibrio vulnificus* recognizes the ligand using an “induced fit” model (Serganov et al., 2004). NMR and biochemical data suggested that in the absence of ligand, the Mg^2+^-induced folded state of the *add* aptamer is largely pre-organized with the interaction between peripheral loops present at the end of stems P2 and P3 already formed but with the ligand-binding pocket remaining disordered (Noeske et al., 2005; Wickiser et al., 2005; Gilbert et al., 2006) These studies suggested that ligand binding induces the transition from this open state to a locally organized conformation. To investigate this further, three *add* aptamer constructs were engineered, two involved 2AP substitutions at position 48 (U48AP) and position 24 (A24AP), both near the binding site (Figure 2D). The third construct contained a 2AP substitution at a more distant position (U35AP) located at the P2-P3 loop-loop interface (Lemay et al., 2006). Upon addition of adenine ligand, position U48AP showed a marked increase in 2AP fluorescence indicating that this nucleotide becomes unstacked and more exposed to the solvent when the ligand binds. While U48AP showed very little change in fluorescence in the presence of Mg^2+^ ions alone, the quenching observed for A24AP was predominantly induced by the addition of Mg^2+^ and indicated that this nucleotide becomes less flexible during folding. For the A35AP variant, one third of the total quenching was observed just after the addition of Mg^2+^ ions, confirming the formation of the loop-loop interaction in the absence of ligand (Lemay et al., 2006; Lemay and Lafontaine, 2007). Interestingly, stopped-flow experiments performed on the three 2AP constructs revealed very similar folding rates (~ 3.1 ± 0.05 × 10^4^ M^−1^ s^−1^; Rieder et al., 2007). This suggested that the organization of the ligand binding pocket monitored using A48AP and A24AP) and the interaction between the peripheral P2 and P3 loops take place at similar time scale and most likely in a concerted manner.

In addition to targeting local changes in nucleotide conformation as described above, 2AP substitution strategies have been used also to investigate long-range conformational changes in natural aptamers (Lang et al., 2007; Blouin et al., 2010; Rieder et al., 2010; Haller et al., 2011a). As an example, 2AP substitutions were used to investigate the influence of Mg^2+^ ions and SAM ligand in the formation of stacking interactions between helical stems of the SAM-I aptamer (Heppell et al., 2011). The SAM-I aptamer is organized around a 4-way RNA junction composed of stems P1–P4 linked through stretches of unpaired nucleotides. Crystallographic data have shown that the ligand-bound state of the SAM-I aptamer organized with the helical stem P1 stacked on the P4 stem and P2 stacked on the P3 helix and both coaxial stacks are oriented with an angle of 70° relative to each other. SAM aptamers carrying the substitutions at positions A71 and A138, which are located at the interface of each stacking unit. The A71AP variant showed a progressive quenching upon addition of increasing concentrations of Mg^2+^ ions but no change was observed with the addition of SAM ligand. In contrast, the A138AP variant displayed a significant quenching with the addition of SAM but no effect was observed with the addition of Mg^2+^ ions in the absence of ligand. From these data, it was concluded that Mg^2+^ ions are critical for the formation of the P2-P3 helical stack and that the coaxial alignment of the P1 and P4 stems depends almost exclusively on ligand binding (Heppell et al., 2011; Eschbach et al., 2012). The application of 2AP substitutions in the context of natural aptamers highlights the potential of this method to report structural changes taking place locally, at the nucleotide level as observed in the adenine aptamer, but also to monitor larger conformational changes, as discussed for the SAM-I sensing aptamer. Because DNA and RNA oligonucleotides are commercially available with 2AP incorporated at the desired positions, and 2AP substitutions do not perturb significantly the nucleic acid structure, they are becoming a widely employed tool to investigate aptamer-ligand complexes.

## Fluorescence methods based on doubly labeled aptamers

The positioning of two covalently attached probes, either two fluorophores or a fluorophore and a quencher, onto an aptamer structure explores the “communication” between both probes and how this is influenced by aptamer folding or ligand binding (Juskowiak, 2011). The term “communication” refers to any photophysical process that takes place between both probes and it is sensitive to the distance that separates them. The majority of assays based on doubly labeled aptamers have used either fluorescence quenching or fluorescence resonance energy transfer (FRET) as the distance-dependent mechanism responsible for modulating the fluorescence output (Nutiu and Li, 2005; Juskowiak, 2011; McCluskey et al., 2014; Boudreault et al., 2015). A doubly-labeled aptamer that explores the concept of quenching between a fluorescence probe and a quencher group is normally referred as a molecular beacon (MB) structure (Tyagi and Kramer, 1996). In addition to quenching and FRET-based mechanisms, the ability of some fluorescence molecules to form dimers when two of them come into close contact, either in the excited-state (excimers) or in the ground-state (exciplexes), and emit at longer wavelength has also been explored (Conlon et al., 2008). In the next section, we will detail the use of quenching/de-quenching and FRET-based molecular beacons to investigate aptamer-ligand complexes.

### Aptamer beacons based on quenching/de-quenching

**Figure 3:**
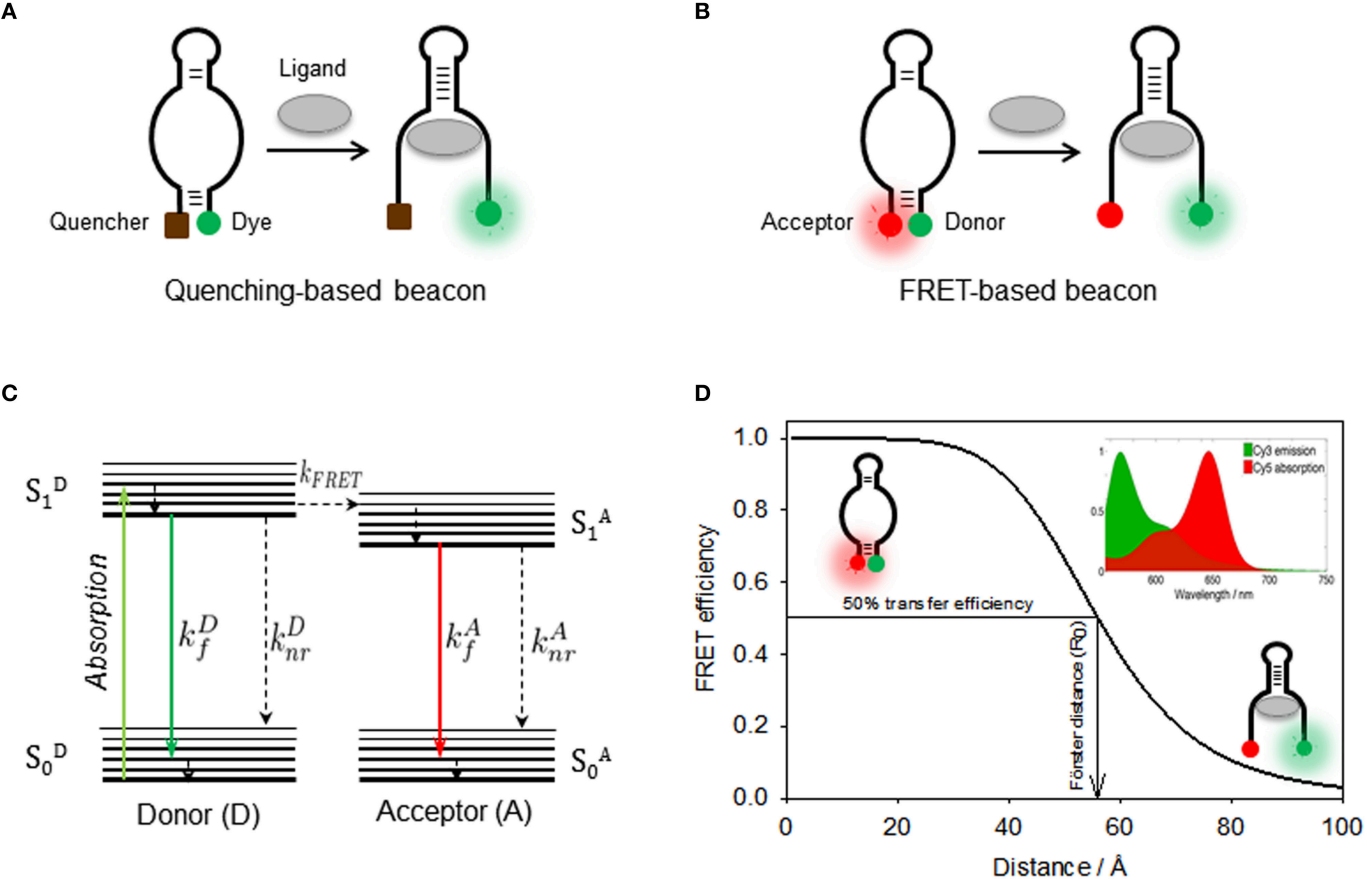
**(A)** Quenching-based and FRET-based **(B)** aptamer beacon strategies to monitor conformational changes of the aptamer structure upon ligand binding. In the FRET-based beacon strategy, the quencher is replaced by a FRET acceptor dye. **(C)** Jablonski diagram showing the principle of non-radiative energy transfer between a donor and a nearby acceptor. Donor is optically excited and reaches its first electronic excited state ${\text{S}}_{1}^{D}$, from which it can then emit fluorescence ($k_{f}^{D}$), relax through non-radiative processes ($k_{nr}^{D}$), or undergo an energy transfer process to the acceptor (*k*_*FRET*_). Then, the acceptor on its ${\text{S}}_{1}^{A}$ state relaxes via a radiative ($k_{f}^{A}$) or a non-radiative ($k_{nr}^{A}$) process. **(D)** Schematics of the use FRET as a molecular ruler to monitor distance changes in a FRET aptamer beacon. The position of the Cy3 donor (green) and the Cy5 acceptor (red) is also shown. FRET efficiency as a function of the donor-acceptor inter-dye distance for the Cy3(D)-Cy5(A) FRET pair, which depends inversely on the sixth power of the distance between both dyes. The distance for which the FRET efficiency reaches 50% is known as Förster radius, which is ~53 Å for the Cy3-Cy5 FRET pair. (Inset) Cy3 emission (green) and Cy5 absorption (red) spectra overlap, which has a direct dependence on the FRET efficiency.

A quenching-based molecular beacon is a synthetic nucleic acid structure containing a stem and a loop region with a fluorophore and a quencher positioned at each end of the stem duplex (Figure 3A; Drake and Tan, 2004; Goel et al., 2005; Stobiecka and Chalupa, 2015). The working principle of a molecular beacon is a structural transition between the mentioned hairpin loop conformation that implies complete quenching of the fluorophore and an alternative structure where binding of the target forces the hairpin loop into an open conformation that increases the distance between both probes and triggers a fluorescence increase (Tyagi and Kramer, 1996; Goel et al., 2005; Zheng et al., 2015).

One of the first examples of merging the molecular beacon concept with aptamer design to generate an aptamer beacon was developed by Ellington and coworkers (Hamaguchi et al., 2001). In order to force the formation of a closed hairpin-loop structure in the absence of target molecule, one end of a thrombin quadruplex aptamer was extended by 4–6 base pairs. It was reasoned that increasing the length of the 5′ end of the thrombin aptamer would cause the aptamer to form the hairpin loop instead of the G-quartet structure and that addition of thrombin stabilizes the latter, thus separating the fluorophore-quencher pair and resulting in a fluorescence increase. In this study the 5′ end of the aptamer was labeled with a fluorescein derivative (FAM) and the 3′ end was tagged with 4-[[4′-(dimethylamino)phenyl]azo]-benzoic acid (Dabcyl) acting as quencher. The fluorescence response of the thrombin aptamer extended by five base pairs showed a 2.5-fold increase at saturating thrombin concentrations and had an apparent dissociation constant of ~10 nM. This is a standard “turn-on” approach based on target recognition inducing a fluorescence enhancement (Wang et al., 2011).

An aptamer beacon strategy to detect thrombin binding was also developed by the Tan's group (Li et al., 2002). Here, the intrinsic equilibrium present in the absence of protein between a random coil and the quadruplex state was used, without the need to force the ligand-free aptamer into a hairpin loop conformation. The thrombin aptamer was modified to act as an aptamer beacon by incorporating a fluorophore (fluorescein) and a quencher (Dabcyl) at each end of the aptamer. A significant quenching of the fluorescein emission signal (~60%) was observed in the presence of the thrombin target as expected for a “turn off” aptamer. An alternative construct was designed in a FRET format. For this, the quencher was replaced by coumarin acting as FRET donor to the acceptor fluorescein was also explored in the same study. The values obtained for the dissociation constant and the detection limits obtained from both constructs were very similar and in the region of 4–5 nM and 350–450 pM. The application of a similar aptamer beacon strategy to sense cocaine, L-argininamide, and ATP-binding aptamers has been recently reviewed (Zheng et al., 2015). A slightly different approach to these fluorophore-quencher strategies was developed and applied to the PDGF aptamer (Yang et al., 2005). In this case, the aptamer was labeled with two pyrene molecules at both ends in such a way that both pyrene groups are separated in the absence of target—and emit in the blue—but become close enough to form an excimer that emits green fluorescence upon PDGF binding. This strategy was also employed with the thrombin and anti-ATP DNA aptamer among others (reviewed in Zheng et al., 2015).

A recently reported strategy explored the concept of contact-induced quenching of an RNA-bound fluorophore for *in vivo* RNA imaging (Sunbul and Jäschke, 2013). Aptamers that selectively bind certain fluorophores such as fluorescein, sulforhodamine B, 5-carboxyetramethylrhodamine (TAMRA), malachite green and rosamine have been developed to image RNA in living cells (reviewed in Urbanek et al., 2014). However, not many applications were found due to high background fluorescence. In an attempt to overcome this limitation, Sunbul and Jascke hypothesized that by covalently coupling the fluorophore to a quencher through a flexible linker so that they can form a close-contact interaction that completely turns off the emission, it would be possible to decrease the background for cellular applications. In the presence of the fluorophore-binding aptamer, this interaction should be more stable to efficiently disrupt the fluorophore-quencher complex and light-up the fluorophore. This concept was tested using as a model the SBR-2 RNA aptamer that binds sulforhodamine with a dissociation constant of ~0.3 μM. Of the tested quenchers, the sulforhodamine-dinitroaniline (SR-DN) pair joined by 2 ethylenglycol units, showed the highest fluorescence enhancement (~100-fold) upon binding to SBR-2 and a dissociation constant ~5-fold higher than the unmodified fluorophore. The formation of a ground-state complex between the dye and DN as being responsible for the quenching mechanism was confirmed from the observation of contact-induced characteristic shifts in the absorbance maximum for the SR-DN pair (574 nm) compared to 568 nm for a sulforhodamine dye carrying only the amino linker (SR-NH2). The quantum yield of the SR-DN molecule bound to the SBR-2 aptamer was 0.65, which is 25 times higher than that of the unbound probe and twice that of SR-NH2, suggesting that the bound fluorophore exhibits a more restricted rotational freedom inside the RNA binding pocket that, presumably, increases its quantum yield. The utility of the SRB-2 aptamer combined with SR-DN in biological applications was confirmed first by monitoring *in vitro* transcription in real-time and then by imaging the SRB-2 aptamer in live *E. coli* using the SR-DN probe (Sunbul and Jäschke, 2013; Arora et al., 2015).

All the aptamer beacons described above employ the full aptamer sequence. An alternative approach based on splitting the aptamer sequence in two fragments that only come together upon ligand binding has also been tested. In an example Stojanovic et al., the aptamer sequence that recognizes the Tat protein of HIV was split in two parts (Stojanovic et al., 2000). One half of the aptamer was engineered to adopt, in the absence of the Tat protein, a hairpin loop conformation that brings in close proximity the fluorophore and the quencher. In the presence of the Tat protein, the two fragments self-assembled resulting in an increase in the distance between both probes that resulted in a 14-fold fluorescence enhancement. In another study, Yamamoto et al. engineered a similar assay but in an array format with one biotinylated fragment immobilized on a streptavidin-coated surface and the other carrying the fluorophore free in solution (Yamamoto et al., 2000). Upon addition of Tat, fluorescence emission from the surface was observed indicating the disruption of the quenched hairpin conformation. Fragmentation of the aptamer in two components was applied also to the cocaine and ATP aptamers and recent developments in the use split-aptamer strategies have been reviewed elsewhere (Heemstra, 2015).

### FRET-based aptamer beacons

A FRET-based aptamer beacon shares many features in common with aptamer beacons based on the fluorescence-quencher approach, but here the quencher has been replaced by a FRET acceptor, a molecule that can emit fluorescence upon non-radiative transfer of energy from the light-excited donor (Figure 3B; Förster, 1948; Blouin et al., 2009b; McCluskey et al., 2014). Fluorescent dyes that constitute suitable FRET pairs such as Fluorescein-Cy3 or Cy3-Cy5 are commercially available with the same chemical groups than the dark quenchers for post-synthetic labeling of nucleic acids. The key advantage of FRET-based beacons is that instead of relying on intensity emission from a single fluorophore, the FRET assay measures the emission ratio between the two fluorescent species, donor and acceptor, and therefore it is more robust against background and other interference sources (Cho et al., 2009). For signaling purposes, because the acceptor emission is at longer wavelength that the acceptor, FRET assays can be considered in the same group than other wavelength-shifting assays, such as those based on pyrene excimer emission (Conlon et al., 2008; Cho et al., 2009). However, whereas pyrene excimer formation requires the close contact between the two dyes to form a dimer, FRET assays are sensitive in a much larger range of inter-dye distances (2–7 nm) (Zadran et al., 2012).

An example about the use of FRET to engineered an aptamer beacon was described for angiogenin (Ang), a 14.4 kDa polypeptide homolog of ribonuclease A (RNase A) that is related to the growth and metastasis (Li et al., 2008). A 45 nt Ang aptamer was engineered to incorporate at the two ends carboxyfluorescence (FAM) acting as donor and tetramethylrhodamine (TMR) acting as acceptor. The aptamer exists in an equilibrium between a non-structured random coil and a secondary structure resembling a stem-loop which is critical for Ang binding. Upon real-time addition of angiogenin, an increase in FRET efficiency was observed indicative of the shortening of the FAM-TMR distance. The FRET signal was stable within 1 min. The FRET assay remained highly specific and sensitive even in a background of other plasma proteins such as HAS, IgG, actin, Thr, and RNaseA. Importantly, the detection limit (2 × 10^−10^ mol L^−1^) meets the requirements for clinical testing of Ang.

A recent report has described the application of FRET-based aptamer beacons for live-cell imaging of polymerase II transcriptional activity. The strategy, so-called IMAGEtags (Intracellular MultiAptamer Genetic tags), involves the use of strings of repeated RNA aptamers engineered to recognize ligands fluorescently labeled with Cy3 and Cy5 dyes (Shin et al., 2014; Ilgu et al., 2016). The Cy3–Cy5 is a widely used FRET pair, where Cy3 acts as the FRET donor and Cy5 as the FRET acceptor. Binding of these exogenous fluorescent ligands to aptamers located next to each other is monitored as an increase in FRET signal. The aptamer string is incorporated into an endogenous gene as a fusion construct or inserted as a synthetic coding region after a promoter of choice for plasmid base expression. The IMAGEtags system may offer some advantages over the other two most prominent mRNA imaging systems, the Spinach RNAs and the MS2 system. The MS2-GFP imaging system is based on the accumulation of fluorescent fusion proteins over the tagged RNA and measures the redistribution of fluorescence signal over the entire cell to localize the reporter RNA, with no change in signal per cell upon increased gene expression (Urbanek et al., 2014). In contrast, IMAGEtags use a change in FRET signal that allows to quantify transcription levels per cell per time. The use of FRET to obtain a high signal/noise ratio and therefore high sensitivity is an additional advantage over Spinach-based detection that relies on intensity changes between the free and bound ligand. Cy3 and Cy5 fluorescently labeled tobramycin and 2-[[(3-Aminophenyl)-methyl]amino]-6-(2,6-dichlorophenyl)-8-methyl-pyrido(2,3-d)pyrimidin-7(8H)-one (PDC) ligand showed excellent reporter activity and no toxicity after 4 h incubation was observed in yeast cells (Shin et al., 2014).

### FRET as a molecular ruler to investigate aptamer structure

Aptamer beacons discussed in the previous section employed the FRET signal as digital readout to report the formation or disruption of the aptamer-ligand complex. However, the high-sensitivity of the energy transfer process with the distance between the donor and the acceptor has been widely employed as a method to provide structural information about the aptamer structure (Blouin et al., 2009b; Zheng, 2010). In the following section we will explain the use of FRET as a molecular ruler and provide some examples about its application, first at ensemble level and then in single-molecule format, to extract information about the structural dynamics of aptamer-ligand interactions. As we briefly mentioned, FRET is a non-radiative process whereby a donor fluorophore (D) in its excited-state transfers energy to a ground-state acceptor (A) as a result of a through-space coupling of their transition dipoles (Figure 3C). The theory behind dipole-dipole coupling has been described in detail in several reviews (Clegg, 1992; Lakowicz, 1999; Zheng, 2006) and it has been demonstrated that the efficiency of the energy transfer process (E) depends on the inverse sixth power of the D-A distance following the expression below:

$$\begin{array}{l} E=\frac{R_{0}^{6}}{R_{0}^{6}\;+\;R^{6}}=\frac{1}{1+{\left(\frac{R}{R_{0}}\right)}^{6}} \end{array}$$

In this equation, *R* is the D-A distance in the biomolecule and *R*_0_ represents the D-A at which the efficiency of the energy transfer process reaches 50% (*E* = 0.5), which is a characteristic parameter for each donor-acceptor combination of fluorophores (Figure 3D). The variables on which the FRET efficiency depends and how to evaluate the Ro value for a given pair in a given biomolecule is beyond the scope of this report, thus we refer the reader to specialized reports for specific details (Zheng, 2006; Blouin et al., 2009b). Several methods have been described to calculate the FRET efficiency when performing experiments with donor and acceptor labeled constructs at ensemble level in a conventional steady-state fluorimeter and how to extract values of D-A distance (Clegg, 1992; Lilley and Wilson, 2000). Among these methods, calculating the ratioA parameter provides the most accurate determination of the FRET efficiency from a steady-state measurement. The ratioA method is based on calculating the amount of acceptor emission due to FRET with respect to the amount of acceptor emission obtained by direct excitation. It has been demonstrated that using the ratioA method minimizes the influence of fluorescence quenching or enhancement process competing with the energy transfer mechanism that can affect the FRET efficiency values and the calculated D-A distance (Blouin et al., 2009b; Hutton et al., 2010; Perez-Gonzalez et al., 2016). A detail description of the ratioA method and how to implement it for the characterization of nucleic acids has been discussed previously (Clegg, 1992; Blouin et al., 2009b; McCluskey et al., 2014).

The application of ensemble FRET to characterize aptamer structure in solution and how this is influenced by metal ions and ligand binding is exemplified by an early study on the adenine riboswitch aptamer (Lemay et al., 2006). In this study, the *add* adenine aptamer from *Vibrio vulnificus* was labeled with a Fluorescein donor and a Cy3 acceptor at positions U27 and U53 located in the P2 and P3 stem loops, respectively. To generate this construct, the aptamer sequence was split in two synthetic strands, each of them carrying an internal amino-allyl uridine modification to which conjugate, after solid-phase synthesis, a succinimidyl ester derivative of the corresponding dye. After labeling and purification of each individual strands, these were ligated using T4 RNA ligase and the full length RNA aptamer was separated using polyacrylamide gel electrophoresis. The FRET efficiency was measured as a function of the concentration of divalent metal ions (Mg^2+^) and adenine ligand. The FRET efficiency progressively increased from a value of E ~ 0.3 to a value of E ~ 0.6 with the addition of Mg^2+^ ions and reached a plateau above 1 mM concentration. These results indicate that the distance between the P2 and P3 decreases with the addition of Mg^2+^. A fit of the FRET isotherm to a two-state model gave values of 22 μM for the magnesium ion concentration at which the FRET transition is 50% complete ([Mg^2+^]_1∕2_) and a Hill coefficient of *n* = 1, which suggested a non-cooperative binding of Mg^2+^ ions. When the FRET titration was repeated in a background of 50 μM adenine ligand, a transition between similar very similar FRET values was observed with a slight decrease in [Mg^2+^]_1∕2_ Overall, these data confirmed that the adenine aptamer is largely pre-organized in the absence of ligand and that the loop-loop interaction is formed at physiological concentrations of divalent metal ions in the absence of ligand.

Although, the guanine and adenine aptamers share a very similar tertiary structure, they exhibit a very high selectivity toward their cognate ligands. It was shown that the specificity of the adenine aptamer for its ligand arises from the formation of a Watson-Crick interaction between the adenine ligand and residue U65, which in the case of the guanine aptamer takes place via residue C74 (Mandal et al., 2003; Mandal and Breaker, 2004). Thus, the discrimination between both ligands by an otherwise structurally similar aptamer relies on the specific nature of a single nucleotide. Using sequence alignment, it was proposed that in addition to this nucleotide, the nature of the residue at position 39 in the adenine aptamer (C, G, or U but no A) or the equivalent residue 48 in the guanine aptamer (A, C, or U but no G) was critical for aptamer function. Thus, sequences such as A39-U65 in the adenine aptamer and G39-C65 in the guanine aptamer have been evolutionary excluded because they severely compromise ligand recognition. The rationale for the inhibitory effect on ligand binding observed for the G39-C65 sequence was confirmed by X-ray crystallography and NMR data (Delfosse et al., 2010) and ensemble FRET (Tremblay et al., 2011). The crystal structure of the G39-C65 variant revealed a complex network of interactions within the aptamer core similar to that of the wild-type aptamer in complex with the ligand. Here, the G39 residue established hydrogen-bond interactions with C65 and thus mimics and replaces the natural ligand leading to a riboswitch that is constitutively activated. An ensemble-FRET analysis of the formation of the loop-loop interaction revealed that the G39-C65 variant exhibits a similar transition to a high FRET state induced by Mg^2+^ ions (Tremblay et al., 2011). However, the FRET values were consistently higher suggesting that the docking of the P2 and P3 loops is a more efficient process for the variant than for the ligand-free wild type aptamer. Because the wild-type aptamer has been shown to fold more efficiently in the presence of adenine ligand by stabilizing the loop-loop interaction, this was interpreted as evidence that the formation of the G39-C65 interaction plays a similar role as the adenine ligand activating the folding process (Tremblay et al., 2011).

Ensemble-FRET techniques have also been applied to monitor more local structural rearrangements such as helical stacking. For instance, the formation of the P1-P4 and P2-P3 helical stacks in the SAM-I riboswitch was also addressed using ensemble-FRET (Heppell et al., 2011; Eschbach et al., 2012). In this case, the SAM-I riboswitch aptamer was engineered from three strands, a transcribed strand and two synthetic RNA sequences carrying the amino modifications required to incorporate the fluorescein donor and the Cy3 acceptor dyes. The global folding of the SAM-I aptamer as a function of Mg^2+^ ions and SAM ligand was analyzed using three FRET vectors that monitor conformational changes involving stems P1 and P3, P1 and P4, and P3 and P4. The distance between the P1 and P3 stems decreased upon addition of Mg^2+^ ions but remained unaltered upon addition of 5 μM SAM ligand. By fitting the FRET isotherm to a two-state Hill model, values of 0.94 mM for half-magnesium concentration and a Hill coefficient of 3.9 were obtained, suggesting that the influence of Mg^2+^ ions in this transition is highly cooperative. A similar analysis revealed that Mg^2+^ ions do not influence the positioning of stems P1 and P4 but their relative distance decreases upon addition of SAM ligand (K_D_ ~ 5 nM). For the remaining FRET vector, P3-P4, the observed behavior consisted of a combination of those observed for P1-P4, with an increase in FRET efficiency with the addition of Mg^2+^ ions and a decrease induced by ligand binding. Taken together, these data were interpreted as evidence for a two-step folding process in which Mg^2+^ and ligand binding induce a distinctive conformational transition. According to this model, which is supported by the 2AP studies described in previous sections (Heppell et al., 2009), Mg^2+^ ions induce the coaxial stacking between P2 and P3 stems and the close juxtaposition between P1 and P3 stems, whereas ligand binding promotes the coaxial stacking of P1 and P4 stems. It was further demonstrated using comparative gel electrophoresis that the formation of the P1-P4 helical stack induced by ligand binding requires the rotation of the P1 stem along its helical axis (Heppell et al., 2011).

### Single-molecule FRET studies of aptamer structural dynamics and ligand binding kinetics

In ensemble-FRET methods, the observed changes in distance represent a statistical average at equilibrium over many molecules and time. In contrast, single-molecule FRET monitors distance values, aptamer by aptamer, and thus information that would be lost due to averaging can be retrieved (McCluskey et al., 2014; Savinov et al., 2014; St-Pierre et al., 2014). For instance, for a given aptamer present in an equilibrium between different structures, sm-FRET allows to determine the number and relative contribution of each of the conformers even when they are low-populated species. This is particularly important in the context of aptamer-ligand interactions, because it provides a mean to investigate whether the formation of the aptamer-ligand complex modifies the structural landscape of the aptamer, as expected for an induced-fit model. Alternatively, it can also selectively binds one of the aptamer conformers present in solution as it would be expected for a conformational selection ligand-binding mechanism (Haller et al., 2011b).

**Figure 4:**
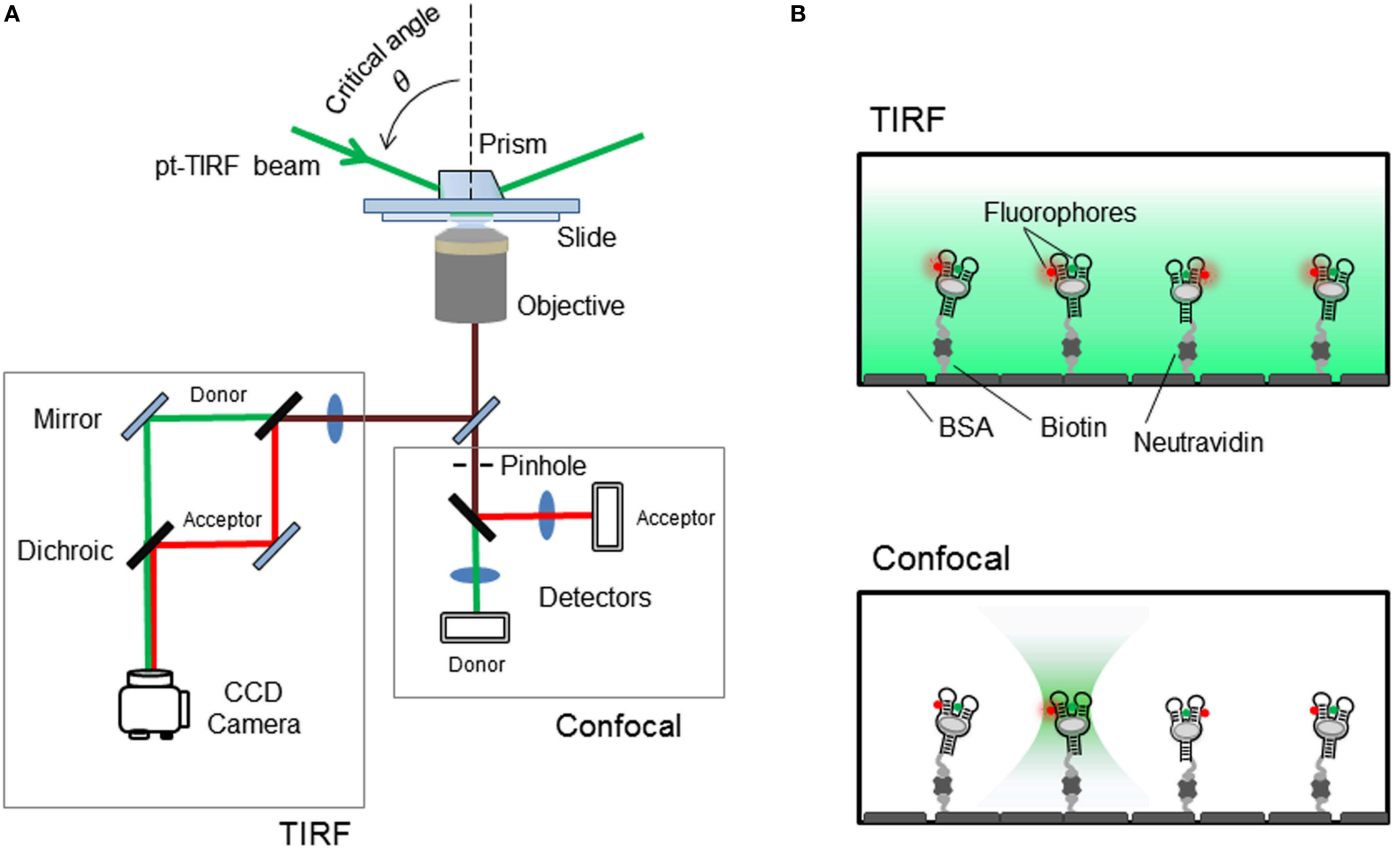
**(A)** Schematics of the single-molecule FRET setup. Detection of the fluorescence emission on a TIRF microscope is collected on a CCD camera, whilst confocal techniques use avalanche photodiodes for faster acquisition times. **(B)** Surface-immobilized wide-field and confocal techniques. Aptamer is immobilized via biotin-avidin interactions. (Top) TIRF is a wide-field technique in which a laser incident at a critical angle generates an evanescent wave on the sample chamber to simultaneously excites hundreds of immobilized and fluorescent-labeled aptamers. The intensity of the evanescent wave follows an exponential decay with a penetration depth of ~150 nm. (Bottom) In confocal microscopy a single immobilized aptamer is excited. The excitation laser is focus to diffraction limit and the resulting fluorescence is collected through a pinhole (~50–100 nm) to prevent out-of-focus light reaching the detector. In both configurations, the fluorescence collected by the high numerical aperture objective (water-immersion for TIR and oil-immersion for confocal) is separated in its donor and acceptor contributions using a dichroic mirror and individually imaged onto a CCD camera (TIR) or onto two avalanche photodiodes (confocal).

Over the last decade, a number of different microscopy techniques have been adapted for FRET studies and have been extensively applied to investigate the structural dynamics of nucleic acid aptamers and other biomolecules (McCluskey et al., 2014). Broadly, these techniques can be grouped on those that immobilize the aptamer on the surface of a microscope slide and those that interrogate the FRET-labeled aptamer whilst it diffuses freely in solution. Although, the immobilization approach requires the introduction of an additional functional group for attachment to the surface, it has been the most widely employed single molecule method to monitor nucleic acid dynamics and protein-nucleic acid interactions. By immobilizing a FRET-labeled aptamer onto the microscope slide it is possible to monitor structural changes from milliseconds to seconds or even minutes, and how these are modulated by solution conditions and ligand binding. The simplest method for aptamer immobilization takes advantage of the strong interaction between biotin and streptavidin (Roy et al., 2008; Blouin et al., 2009b). For this, the aptamer is modified with the biotin group and the microscope slide is coated with streptavidin. Visualizing immobilized aptamers has been mostly carried out using a total-internal reflection (TIR) as the excitation method (Roy et al., 2008) although other techniques such as confocal microscopy have also been used (Roy et al., 2008; Perez-Gonzalez and Penedo, 2015), particularly when the biomolecule under investigation exhibits dynamics in the low millisecond regime (<10 ms) (Figures 4A,B).

As shown in Figure 4, to achieve TIR illumination, the excitation light from a continuous laser source is directed at a particular angle, so-called the critical angle, and coupled to the sample slide using a prism. In this configuration, when the beam propagates from the high-refractive index medium (*n* ~ 1.43 for quartz) to the low-refractive index aqueous medium (*n* ~ 1.33), the majority of the beam is reflected at the interface and only a small evanescent wave propagates into the sample (Figure 4B). The intensity of this evanescent wave decays exponentially within 100–200 nm from the interface between the two media and depends on the angle of incidence above the critical angle and the actual wavelength of the light. The use of this thin-layer of illumination allows to confine the excitation light to where the FRET-labeled aptamer molecules are immobilized, thus reducing the noise contributions from scattering and background signals. In a single-molecule FRET experiment using TIR illumination, the fluorescence image of both donor and acceptor is collected by the objective and then split in two light paths that impinge the camera at different locations. The donor emission is directed onto the left half of the camera and the acceptor emission onto the right half of the CCD chip. Thus, for each immobilized aptamer observed on the camera field of view, every fluorescent spot on the donor channel (left) has a mirror image on the acceptor channel (right) and from the fluctuations in the intensity of both signals over time it is possible to extract FRET efficiency value using the expression, E = I_A_/(I_A_+I_D_), that correlates with the conformational change taking place. Here, I_A_ and I_D_ represent the intensity of the acceptor and donor, respectively. Readers interesting in obtaining more information about the technical implementation of single-molecule FRET are encouraged to consult several specialized reviews in the field. The technical aspects of the implementation and calibration of TIR and confocal single-molecule FRET methods have been covered in detail in previous reviews (Roy et al., 2008; Blouin et al., 2009b; Yang et al., 2013; Gust et al., 2014; Deniz, 2016).

**Figure 5:**
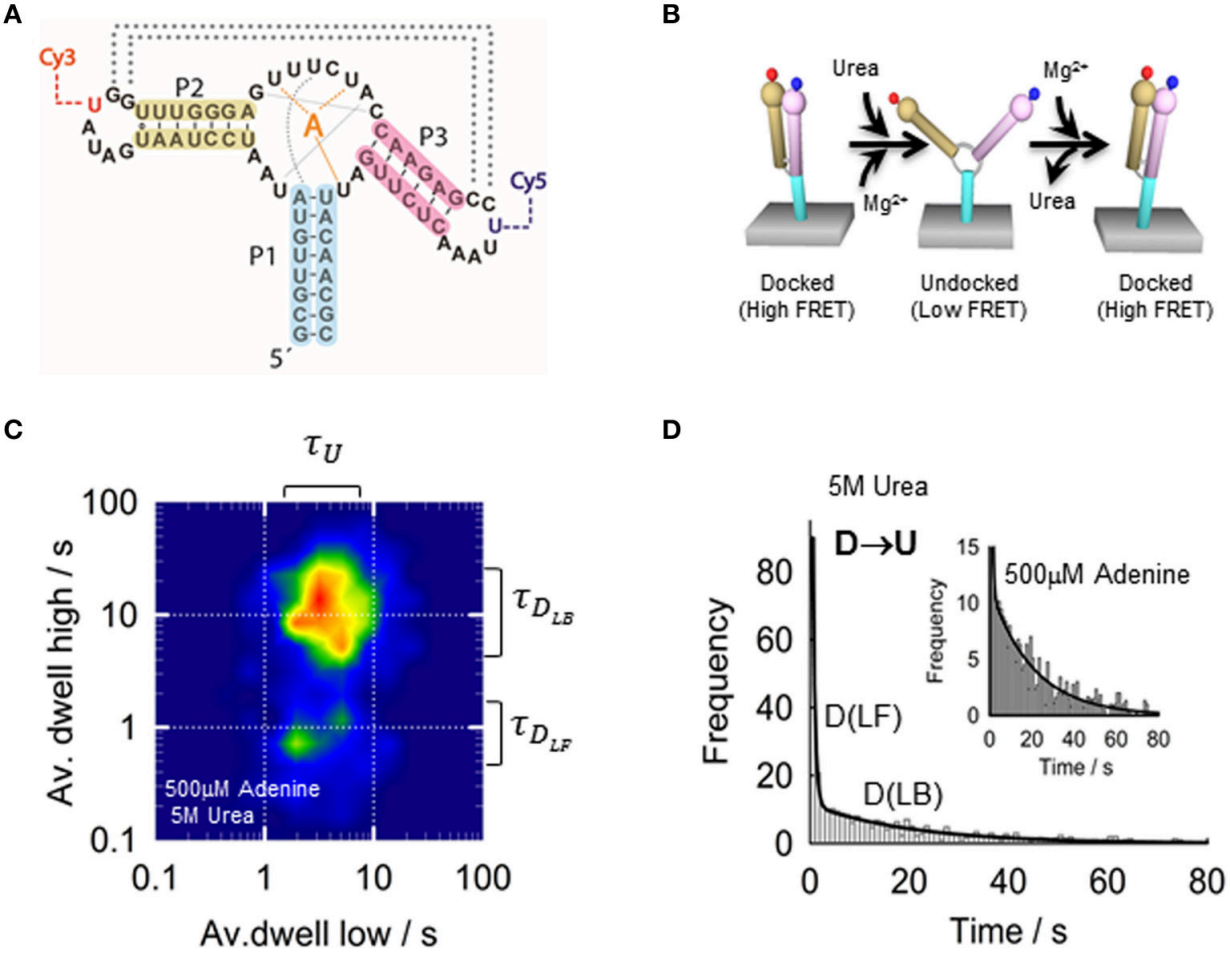
**(A)** Schematic of the adenine riboswitch aptamer showing the residues involved in the ligand binding (orange), stabilization of the native conformation (gray) and P2-P3 loop-loop interaction (dotted lines). Loops P2 and P3 incorporate the labeling positions for the Cy3 and Cy5, respectively. **(B)** Conformational changes of the adenine aptamer representing the docking induced by Mg^2+^ and the undocking induced by urea. **(C)** Two-dimensional contour plot representing the averaged dwell times of the docking and undocking events at 4 mM MgCl_2_, 500 μM adenine 5 M urea. **(D)** Dwell-time histograms for urea-induced undocking fitted to a bi-exponential function (solid line), reporting a value of 2.1 ± 0.1 s^−1^ (ligand bound, D_LB_) and 0.045 ± 0.003 s^−1^ (ligand-free, D_LF_) in the presence of 500 μM adenine and 5 M urea. The difference between both rates suggests a 50-fold stabilization of the aptamer structure upon ligand binding.

In the next section, we will use the *add* adenine aptamer from *V. vulnificus* as a model example on the application of smFRET to an aptamer-ligand complex (Lemay et al., 2006; Tremblay et al., 2011), As previously described for ensemble-FRET, the *add* aptamer was labeled at the P2 and P3 stem loops with a FRET pair (Figure 5A), but for sm-FRET the Cy3–Cy5 combination was used, instead of Fl-Cy3, due to its much higher photostability under continuous excitation with laser light. Single aptamers immobilized on the microscope slide displayed a Mg^2+^-induced transition from a low-FRET state (*E* ~ 0.25) to a high-FRET state (*E* ~ 0.9) as observed in ensemble studies. The low-FRET state was assigned to an aptamer conformation where the loop-loop interaction was not formed (undocked, **U**) and the high-FRET state as resulting from the close interaction between both loops (docked state, **D**). However, sm-FRET revealed the presence of an additional intermediate state (**I**) between **U** and **D**. Analysis of the single-molecule FRET trajectories for many **U** ↔ **D** transitions indicated that the formation of the loop-loop interaction was taking place mostly from the **I** state (**U** ↔ **I** ↔ **D**). This was taken as evidence that the **I** state represented an obligatory conformer in the folding landscape of the add aptamer (Lemay et al., 2006). Interestingly, the addition of adenine ligand had two main effects on the structural dynamics of the aptamer. First, adenine binding to the aptamer stabilized the docked conformation (docked and ligand bound, **D**_LB_) compared to the ligand-free state (**D**_LF_). Second, the relative population of the **I** state for a given concentration of Mg^2+^ ions increased upon addition of the ligand. Stabilization of the aptamer upon ligand binding has been previously observed and it is expected as an integral part of the regulatory process; however, its influence on the formation of the intermediate state is less intuitive and suggests that the ligand can actively modulate the folding landscape of the aptamer by interacting with partially folded aptamers. The influence of the ligand on the structural dynamics of the aptamer became more pronounced when comparing the docking (**U** ↔ **D**) and undocking (**D** ↔ **U**) rates with and without ligand. In the absence of adenine ligand, a high degree of heterogeneity from aptamer molecule to aptamer molecule was observed, with both rates spreading over two orders of magnitude. However, this heterogeneity was greatly reduced for both rates upon addition of saturating concentrations of ligand.

To investigate the formation of the *add* aptamer-ligand complex in further detail, a later report combined sm-FRET with the use of chemical denaturants such as a urea (Dalgarno et al., 2013). Although, chemical denaturants are widely employed in protein folding research at single-molecule level, this constituted the first systematic study demonstrating the feasibility of urea-induced denaturation of the RNA tertiary structure to analyze aptamer-ligand interactions (Shaw et al., 2014). The study demonstrated that surface-immobilized adenine aptamers labeled as previously at the P2 and P3 stem loops with the Cy3–Cy5 FRET pair can be subjected to Mg^2+^-induced folding and urea-induced unfolding cycles without compromising surface-attachment or RNA folding integrity (Figure 5B). Moreover, the study demonstrated that urea-induced disruption of the loop-loop interaction takes place via an intermediate state (**I**) as observed when using Mg^2+^ ions to promote folding. Overall, the study showed how the competing interplay between folding agents such as Mg^2+^ and ligand and unfolding species such as urea can be harnessed to extract information that otherwise remains hidden (Shaw et al., 2014). For instance, aptamers labeled with a FRET pair to monitor the loop-loop interaction between stems P2 and P3 have identical energy transfer efficiency for the ligand-free and the ligand-bound states (*E* ~ 0.9). At 4 mM concentration of Mg^2+^ ions and in the presence of 5 M urea, the addition of 500 μM adenine ligand slowed down the undocking rate from a value of 2 ± 0.01 s^−1^ to a value of ~0.045 ± 0.003 s^−1^ (Figures 5C,D). This 50-fold difference in the undocking rate can thus be used to dynamically differentiate between the ligand-free (**D**_LF_) and ligand-bound aptamers (**D**_LB_) even when they displayed identical FRET value.

Single-molecule TIR-FRET has been employed to investigate other natural aptamers including those that recognize guanine (Brenner et al., 2010), SAM (Heppell et al., 2011; Haller et al., 2011b), lysine (Fiegland et al., 2012), TPP (Haller et al., 2013), preQ1 (Suddala et al., 2013), and c-di-GMP (Wood et al., 2012). The ability of single-molecule TIR-FRET to monitor, and very often, to differentiate conformational changes between the ligand-free and ligand-bound forms has been explored to elaborate models of ligand-binding (Haller et al., 2011b; McCluskey et al., 2014; Savinov et al., 2014). From these studies, it has become clear that the traditional classification of ligand recognition mechanisms according to “induced-fit (IF)” or “conformational selection (CS)” models is an oversimplification and intermediate behaviors between both limiting models are more a rule than an exception (Haller et al., 2011b). For instance, a recent study single-molecule TIR-FRET on the pre-Q1 riboswitch aptamer from *B. subtillis* (*Bsu*), the smallest known aptamer domain with just 34 nt, has provided evidences for Mg^2+^ ions controlling not only folding of the aptamer but also the mechanism of ligand recognition (Suddala et al., 2015). The *Bsu* PreQ1-I riboswitch senses the intracellular concentration of queuosine intermediates preQ1 (7-aminomethyl-7-deazaguanine) and possibly preQ_0_ (7-cyano-7-deazaguanine) to terminate transcription of genes. At very low concentrations of Mg^2+^ ions, the ligand binds to an unfolded hairpin conformation and promotes the formation of a pseudoknot structure that encloses the ligand. This IF mechanism is supported by NMR studies that suggest that the aptamer-ligand complex can form even when the aptamer structure is only partially folded. In contrast, in the presence of Mg^2+^ ions, folded conformations become more populated and captured by the ligand in agreement with a conformational selection process. Interestingly, concentrations of Mg^2+^ ions as low as 9 μM seem to be sufficient to switch from an IF to a CS ligand-binding mechanism (Suddala et al., 2015). Distinguishing between both mechanisms has been challenging using conventional ensemble techniques and this study highlights the potential of single-molecule fluorescence to investigate the intricate relationship between aptamer folding and structure and ligand-binding dynamics.

## Future directions

Artificially-engineered and naturally-occurring aptamers able to recognize specific targets are emerging as an important class of functional nucleic acids with potential applications in many areas of biotechnology. However, our current understanding of the molecular rules by which an RNA sequence is able to efficiently discriminate between structurally related compounds is still in its infancy, and without this knowledge, our ability to harness the full potential of aptamers as nanosensors, as intracellular RNA imaging agents, or as therapeutic elements is severely compromised. Because of the intrinsic dynamic character of the aptamer structure and the ligand-binding process, fluorescence methods, and particularly those based on single-molecule detection, are uniquely placed to provide insights into the molecular properties that dictate the formation of a particular aptamer-ligand complex in solution. However, current fluorescence-based single-molecule methods require the use of extrinsic dyes that are relatively large and therefore limited to positions far from the ligand binding pocket. In this context, the development of single-molecule methods that can use the intrinsic fluorescence signal of nucleotide mimics, such as 2-aminopurine, pyrrolo-dC and other non-natural compounds, that can be incorporated at almost any position within the sequence with minimal disruption of the aptamer architecture will be greatly beneficial. Ideally, single-molecule FRET using extrinsic dyes could target long-range conformational changes taking place between peripheral motifs of the aptamer structure and small molecule nucleotide analogs could be used to monitor structural changes within the binding pocket. The development of FRET assays involving such nucleotide analogs will enable to measure nanometer-size distance changes that to date are only affordable using complex NMR techniques that require very specialized knowledge and significant amounts of material. Finally, regulatory aptamers function mostly during transcription using a delicate balance between transcription rate, ligand-binding and folding dynamics. However, with the exception of a handful of proof-of-concept examples using single-molecule force to monitor co-transcriptional folding, there is no fluorescence-based single-molecule technique to monitor nascent RNA folding and ligand binding during transcription. The development of a method capable of incorporating dyes at specific positions of the nascent RNA chain will allow to investigate the regulatory function of natural aptamers as it takes place in the cell.

## Author contributions

JC, DL designed the contents of the manuscript and the overall layout. JC, DL, and CP wrote the manuscript. CP made the figures and edited the final version.

## Funding

Funding for open access charge: University of St Andrews.

### Conflict of interest statement

The authors declare that the research was conducted in the absence of any commercial or financial relationships that could be construed as a potential conflict of interest.
